# Role of Bead Sequence in Underwater Welding

**DOI:** 10.3390/ma12203372

**Published:** 2019-10-16

**Authors:** Jacek Tomków, Dariusz Fydrych, Grzegorz Rogalski

**Affiliations:** Faculty of Mechanical Engineering, Gdańsk University of Technology, G. Narutowicza Street 11/12, 80-233 Gdańsk, Poland; dariusz.fydrych@pg.edu.pl (D.F.); grzegorz.rogalski@pg.edu.pl (G.R.)

**Keywords:** underwater welding, covered electrodes, wet welding, cold cracking, pad welding

## Abstract

This paper presents examinations of the role of the bead sequence in underwater welding. Two specimens of wet welded layers made by covered electrodes with the use of normalized S355G10+N steel were welded by a reasonable bead sequence. For each specimen, metallographic macro- and micro-scopic tests were done. Then, Vickers HV10 hardness measurements were conducted for each pad weld in the welded layer. The results show that welding in the water environment carries many problems in the stability of the welding arc, which influences the properties of the welds. The effects of refining and tempering the structure in heat-affected zones of earlier laid beads was observed, which provides a reduction of hardness. The possibility of applying two techniques while welding the layer by the wet method is described. It is stated that a reasonable bead sequence can decrease the hardness in heat-affected zones up to 40 HV10. Tempering by heat from next beads can also change the microstructure in this area by tempering martensite and can decrease susceptibility to cold cracking.

## 1. Introduction

The most common method of underwater welding is wet welding. The welder and the welding area are in direct contact with the surrounding environment. The process is often carried out by flux-cored arc welding (FCAW) [1,2,3,4]. The most common is welding by covered electrodes, which is cheaper and easier [5,6]. 

The water environment can generate significant problems during welding. For welders, the biggest problems are instability of the welding arc and limited visibility [7,8], which can result in poor quality of welded joints. From the metallurgical point of view, the high cooling rate, the high hydrogen content in deposited metal, and residual stresses have the greatest influence on the quality of underwater welded joints.

The high cooling rate leads to the formation of hardened structures in the heat-affected zone (HAZ) [9,10]. This is one of the reasons for residual stresses in welded joints after the welding process is completed. The next problem in underwater conditions is high diffusible hydrogen content in deposited metal, which is much higher than welding in air [11]. Three factors are responsible for high susceptibility of underwater welded joints to cold cracking. This type of cracking is located in the HAZ and in welds of joints made in water along the fusion line in the overheated area of the HAZ [6,12]. Sometimes, they can also be found in the welds [13].

Experiments in the field of welding processes in the water environment are developing annually, because underwater processes are used more in engineering [14].

Attempts to reduce the imperfections in welded joints made in the water are made in different ways. Much attention is devoted to improving the stability of the welding arc in underwater conditions and assessing the metal transfer in the welding arc, using the following methods: visual sensing [15], in situ imaging [16], mechanical constraints [17], and ultrasonic waves [3,8]. The other research trend is developing filler materials for welding: modifying the flux coating and wires of coated electrodes [18] and applying a waterproof coating [19]. Several articles present the results of technological methods to improve the weldability of steel: diffusible hydrogen content in deposited metal [11,20], the use of austenitic deposits [9,21], preheating [22], and multilayer welding, including the temper bead welding technique [6,12,13,23]. Controlling the distribution of heat input by controlling the sequence and positioning of the beads is considered to be particularly promising, because it leads to a reduction in the grain size and provides decreased hardness and increased impact strength [24,25,26]. Different variants of bead sequences are used to weld hard-to-weld metals such as cast iron, nuclear steel, aluminum, quenched and tempered steel, and thermomechanically-treated steel [27,28,29,30,31,32,33,34,35].

For marine and offshore constructions, high-strength, low-alloy (HSLA) steels are widely used [36]. These constructions may undergo failure during exploitation in marine conditions [37,38,39,40]. They often have to be repaired in underwater conditions due to the high cost of transport to air conditions. However, the water environment makes repair welding more difficult, therefore, there is still a search for ways to improve the quality of underwater welds.

The aim of the research was to check the influence of the role of bead sequence on the structure and properties of layers using the wet welding method for specimens welded with covered electrodes.

## 2. Materials and Methods

### 2.1. Materials Used

For tests, normalized S355G10 + N steel plates with dimensions of 16 × 100 × 100 mm were chosen as the base metal (BM). This steel is often used as a material for offshore structures that may require repairs in the water environment. The carbon equivalent (Ce_IIW_) is 0.385 according to the International Institute of Welding (IIW). As a filler material, ISO 2560-A: E 38 0 R 11 [41] rutile electrodes with a diameter of 4.0 mm were used. They produce good plasticity of welds, which may be helpful to avoid cold cracking. The chemical compositions of the materials used are listed in Table 1 and the mechanical properties in Table 2.

### 2.2. Welding Process

**Figure 1:**
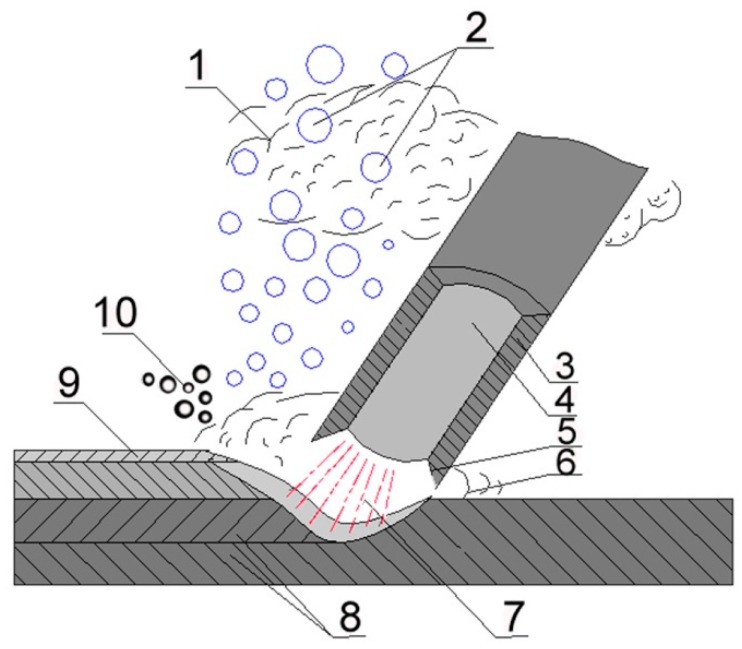
Wet welding by covered electrodes schema. 1: water vapor, 2: gas bubbles, 3: flux coating, 4: wire, 5: melting flux coating, 6: liquid metal, 7: electric arc, 8: base materials, 9: slag, 10: gas from melting flux coating.

For welding, the manual metal arc (MMA) wet welding process was chosen. The schema of this process is presented in Figure 1.

**Figure 2:**
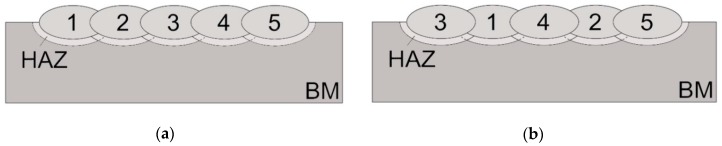
Scheme of welding process in (**a**) specimen S1 and (**b**) specimen S2.

For the tests, 2 specimens were made in the water (0.15 m depth) with 5 beads in each. The time between welding each bead was 120 s. Specimens were welded by different techniques. In sample S1, the beads were laid one after the other from bead 1 to bead 5. In sample S2, the beads were not laid in order. These 2 techniques were chosen because they are representative of welding techniques. In S1, a bead was tempered only by the bead welded later. In S2, beads 1 and 2 were tempered by 2 beads. The use of other techniques, such as 14253, would obtain only one bead (bead 2), which will be tempered by 2 others. That may not have allowed preparation of the planned experiments. The schemas of welding techniques for S1 and S2 specimens are presented in Figure 2. The welding parameters were chosen according to preliminary tests. They provided stability of the welding arc in the experimental conditions and allowed heat input values higher than 0.90 kJ/mm. From the point of view of weldability in underwater conditions, these values may reduce the susceptibility to cold cracking. It has been assumed that the heat input of the tempering bead should be higher than that of the tempered bead. The parameters for both specimens are shown in Table 3. In the case of manual underwater wet welding, the ability to control the value of heat input is very limited. Hence, differences between heat input values of subsequent beads are not significant (0.24 and 0.39 kJ/mm for specimens S1 and S2, respectively) from the point of view of metallurgical transformations.

### 2.3. Examination Procedure

After welding, specimens were cut to cross-sections, and macroscopic and microscopic metallographic examinations were conducted in accordance with the EN ISO 17637:2011 [42] standard, with the use of 4% Nital. Finally, Vickers HV10 hardness measurements were taken in accordance with EN ISO 9015:2011 [43], by using a Sinowon V-10 stand (Sinowon, Dongguan, China), with a measurement error of ± 3 HV10. The investigated S355G10 + N steel is classified as material group 2.1 in accordance with EN ISO 15614-1:2017 [44]. The maximum hardness values of HAZ recommended by this standard cannot exceed 380 HV10. 

**Figure 3:**
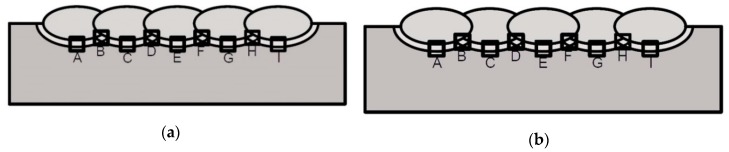
Schematic view of areas of microscopic testing: (**a**) specimen S1, (**b**) specimen S2.

**Figure 4:**
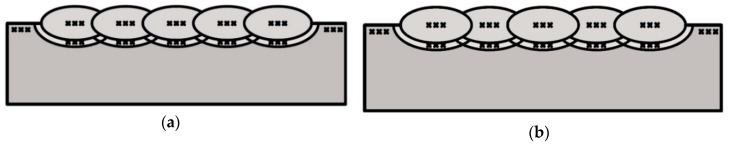
Hardness distribution points: (**a**) specimen S1, (**b**) specimen S2.

Macroscopic tests were prepared for cross-sections of both specimens. Microscopic tests were prepared for the base material in welds and in specific areas of the samples. The first areas were located in the HAZ in the axis of each weld. The second areas were located where the HAZ from the previous weld was overlaid by the HAZ from welds deposited later. Schematic views of the areas of microscopic testing are presented in Figure 3. HV10 hardness measurements were prepared for cross-sections of both specimens in the BM, each weld, and the HAZ of each weld in their axis according to the schemas presented in Figure 4. 

## 3. Results and Discussion

### 3.1. Macroscopic Testing

**Figure 5:**
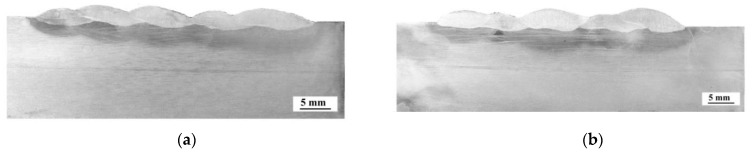
Results of macroscopic testing: (**a**) specimen S1, (**b**) specimen S2.

Macroscopic testing showed that both specimens were welded according to the assumed research plan (Figure 2). No imperfections were found in cross-sections of specimens S1 and S2. The characteristic cross-sectional sizes of all beads had similar dimensions, resulting from small differences in the values of heat input (Table 3). A factor that complicates predicting the effects of multi-pass welding is dilution, which affects, among other aspects, hardenability. Dilution values were calculated with the methodology presented in the literature [45,46]. The cross-sectional area of the fusion zone for each pass was used, i.e., D = A_base_/A, where A is the total molten area and A_base_ is the molten area of the BM [45]. The calculations showed that dilution in S2 is lower than in S1, which may result in the structures and hardness for both specimens [47]. The results of macroscopic testing are presented in Figure 5. The dilution in S1 and S2 is presented in Table 4.

### 3.2. Microscopic Testing

**Figure 6:**
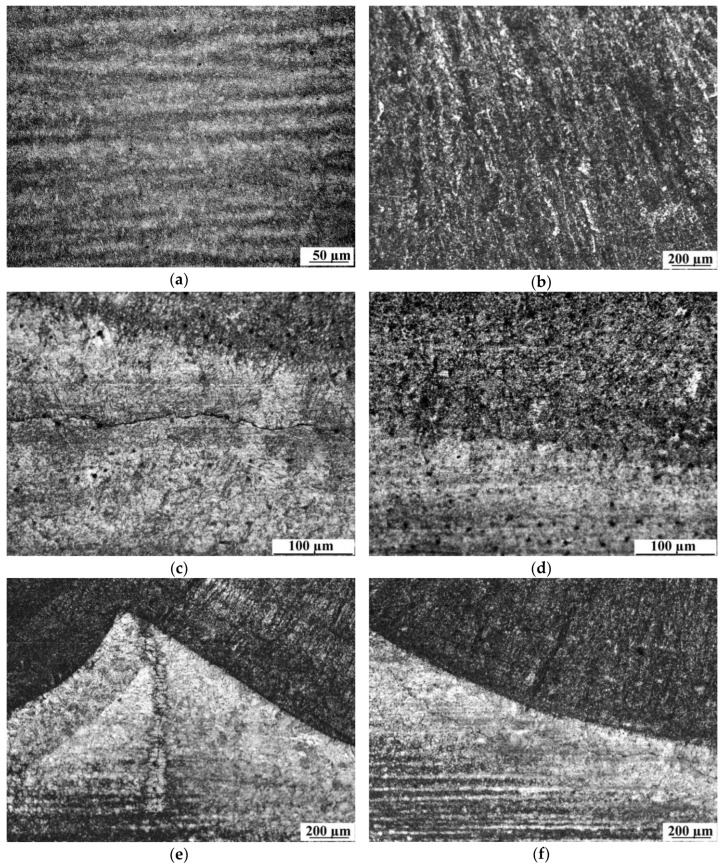
Results of microscopic testing: (**a**) base material, (**b**) weld, (**c**) heat-affected zone (HAZ) in S1, (**d**) HAZ of weld number 1 (area C) in S2, (**e**) overlapping of two HAZs (area H) in S2, (**f**) HAZ of weld 3 (area A) in S2. Etch: 4% Nital.

The results of microscopic testing are presented in Figure 5. Microscopic testing of base material showed that the investigated S355G10 + N steel consisted of fine-grained pearlite and fine-grained ferrite with layers (Figure 6a). The beads consisted of bright fine-grained ferrite arranged in columns, from which grew acicular ferrite at the boundaries of dendrites. Inside them were fine ferrite grains (Figure 6b). These structures are typical for joints made in underwater welding of HSLA steel. In the HAZ of each weld in S1 (regions A, C, E, and G), brittle structures such as bainitic and martensitic were found (Figure 6c). The structures in the HAZ in S2 were the same in areas A, E, and I. In the HAZ of welds 1 and 2 (areas C and G), the structure indicated the presence of refined and tempered low-carbon martensite mixed with normalization structures with fine ferrite and pearlite (Figure 6d), which is a result of the heat influence from welds laid later (Figure 2b). These types of structures have lower susceptibility to cold cracking. The areas where the HAZ of one bead overlapping the HAZ of the bead laid earlier (areas B, D, F, and H) in both specimens are characterized by normalization structures with fine ferrite and pearlite (Figure 6e). While formation and decomposition of austenite occurred twice in regions B, D, F, and H, it happened once in areas A, C, E, and G, followed by in-process tempering with the heat of the next bead. Cracks were found in each HAZ of S1 (Figure 6c), which resulted in brittle structures in this area. In S2, cracks were found only in the HAZ of welds 3, 4, and 5 (Figure 6f) and in area H (Figure 6e). This cracking could have occurred before weld number 5 was laid, which tempered the HAZ of weld 2. Tempering from welds laid later could not repair the microcracks that occurred during the welding of the previous bead and could even propagate these cracks, which is similar to the effect of the temper bead welding technique [12,13]. The location and the length of the cracks in both specimens are summarized in Table 5. The longest cracks were observed in HAZ of welds prepared with the lowest values of heat input.

### 3.3. Hardness Measurements

The investigated S355G10 + N steel is classified as material group 2.1 by EN ISO 9015-1:2011. In accordance with the EN-ISO 15614:1 standard, the maximum hardness of the HAZ cannot exceed 380 HV10 for material group 2.1. The hardness in welds for both specimens was lower than that in the HAZ, which is typical for welding HSLA steel in the water environment [5]. Measurements in the HAZ showed significant differences in hardness distribution in the two tested specimens. The HV10 values in the HAZ were higher in S1 than in S2, which is connected with the different dilution values in both specimens. It is stated in the literature [45], that the higher dilution leads, for example, to more martensite in the welded joint, which is a brittle structure. In S1, most of the measured values did not fulfill the criterion of EN ISO 15614-1 standard of 380 HV10. The welding technique used in S2 allows reduced hardness in the HAZ, especially for welds that have been tempered by heat from welds laid later. The reduction of hardness by welding with the technique used in S2 can reduce the probability of cold cracks occurring in the HAZ. The differences of HAZ hardness are associated with the transformation cycle and tempering effect, which is characteristic for multi-pass welds [48].

**Figure 7:**
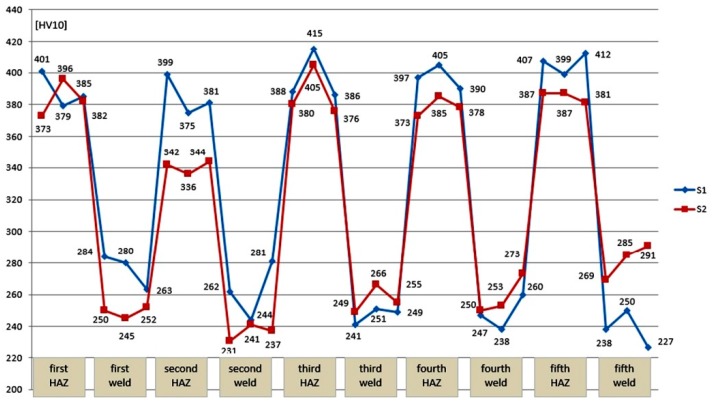
Hardness measurement results for specimens S1 and S2.

The hardness distribution in each point is presented in Figure 7. The results shown in this figure are arranged in order of bead appearance (from left to right) in specimens, e.g., “second weld” means this is the second weld from the left. The average results of hardness measurements in each area are presented in Table 6. 

## 4. Conclusions

The results of the prepared experiments show that the technique used has a significant role in the process of repairs in underwater conditions. The technique used for preparation of specimen S1 can cause a need for further repairs, because it allows cold cracks to form.

The prepared experiments allow us to draw the following conclusions:

1. Water as a welding environment causes cold cracks to occur in the HAZ of wet welded HSLA steel. The susceptibility of steel to cold cracking can be reduced by changing the sequence of the beads during the process.
2. The technique used for the preparation of specimen S2 allowed the structure in the HAZ of some welds to be changed. In this area, the structure indicated the presence of refined and tempered low-carbon martensite mixed with normalization structures with fine ferrite and pearlite. In each HAZ in specimen S1, brittle structures such as bainitic and martensitic were found.
3. Welding S355G10 + N steel with the sequence used in specimen S2 (out of order) allowed a reduction of hardness in the HAZ below the critical value of 380 HV10, as stated by the EN-ISO 15614-1 standard. The lowest hardness values were measured in the HAZ in specimen S2, where the heat from beads laid later tempered this area, and in which the dilution is lower than in specimen S1.
4. The technique used in welding specimen S2 should be used during repair procedures that have to be prepared in the water environment.

## Figures and Tables

**Table 1:** Chemical compositions of materials used (wt.%).

Material	C	Si	Mn	P	Cr	Mo	Ni	Cu	V	Ce_IIW_
S355G10 + N according to control analysis	0.11	0.35	1.39	0.01	0.02	0.02	0.25	0.27	0.003	0.385
E 38 9 R 11 electrode deposit according to manufacturer data	0.07	0.44	0.55	0.01	0.04	-	-	0.05	-	-

Ce_IIW_, carbon equivalent by International Institute of Welding.

**Table 2:** Mechanical properties of materials used according to manufacturer data.

Material	Re (Mpa)	Rm (Mpa)	A_5_ (%)
SS355G10+N	389	521	23.5
E 38 0 R 11 electrode deposit	503	538	26

**Table 3:** Welding parameters for specimens S1 and S2.

Bead No.	I (A)	U (V)	Vsp (mm/s)	Ql (kJ/mm)
Specimen S1				
1	220	21.8	5.95	1.03
2	216	29.5	6.17	1.03
3	224	26.8	5.88	1.02
4	220	29.0	5.38	1.19
5	220	28.8	4.97	1.27
Specimen S2				
1	220	28.8	6.33	1.00
2	216	30.3	5.85	1.12
3	220	28.8	5.88	1.05
4	216	30.0	5.41	1.20
5	216	29.0	4.58	1.39

**Table 4:** The dilution in the S1 and S2.

S1	D (%)	S2	D (%)
Weld1	0.54	Weld3	0.32
Weld2	0.37	Weld1	0.45
Weld3	0.40	Weld4	0.28
Weld4	0.59	Weld2	0.39
Weld5	0.44	Weld5	0.26

**Table 5:** The length of cracks in specimens S1 and S2.

Area (Figure 3)									
Specimen	A	B	C	D	E	F	G	H	I
S1	550 µm	none	350 µm	100 µm	350 µm	none	250 µm	none	200 µm
S2	350 µm	none	none	none	200 µm	none	none	600 µm	150 µm

**Table 6:** Average HV10 hardness measurements results.

Specimen	BM	HAZ 1	Weld 1	HAZ 2	Weld 2	HAZ 3	Weld 3	HAZ 4	Weld 4	HAZ 5	Weld 5	BM
S1	175	388	276	385	262	396	247	397	248	406	238	188
S2	173	384	249	340	236	257	256	379	259	385	282	176
